# Evaluation of HbA1c from CGM traces in an Indian population

**DOI:** 10.3389/fendo.2023.1264072

**Published:** 2023-11-20

**Authors:** Sayantan Majumdar, Saurabh D. Kalamkar, Shashikant Dudhgaonkar, Kishor M. Shelgikar, Saroj Ghaskadbi, Pranay Goel

**Affiliations:** ^1^ Department of Biology, Indian Institute of Science Education and Research Pune, Pune, Maharashtra, India; ^2^ Department of Zoology, Savitribai Phule Pune University, Pune, Maharashtra, India; ^3^ Health Centre, Savitribai Phule Pune University, Pune, Maharashtra, India; ^4^ Department of General Medicine, Joshi Hospital, Pune, Maharashtra, India

**Keywords:** continuous glucose monitoring (CGM), glycated hemoglobin (HbA1c), type 2 diabetes (T2D), average blood glucose concentration (aBG), average interstitial fluid glucose concentration (aISF)

## Abstract

**Introduction:**

The development of continuous glucose monitoring (CGM) over the last decade has provided access to many consecutive glucose concentration measurements from patients. A standard method for estimating glycated hemoglobin (HbA1c), already established in the literature, is based on its relationship with the average blood glucose concentration (aBG). We showed that the estimates obtained using the standard method were not sufficiently reliable for an Indian population and suggested two new methods for estimating HbA1c.

**Methods:**

Two datasets providing a total of 128 CGM and their corresponding HbA1c levels were received from two centers: Health Centre, Savitribai Phule Pune University, Pune and Joshi Hospital, Pune, from patients already diagnosed with diabetes, non-diabetes, and pre-diabetes. We filtered 112 data-sufficient CGM traces, of which 80 traces were used to construct two models using linear regression. The first model estimates HbA1c directly from the average interstitial fluid glucose concentration (aISF) of the CGM trace and the second model proceeds in two steps: first, aISF is scaled to aBG, and then aBG is converted to HbA1c via the Nathan model. Our models were tested on the remaining 32 data- sufficient traces. We also provided 95% confidence and prediction intervals for HbA1c estimates.

**Results:**

The direct model (first model) for estimating HbA1c was HbA1c_mmol/mol_ = 0.319 × aISF_mg/dL_ + 16.73 and the adapted Nathan model (second model) for estimating HbA1c is HbA1c_mmol/dL_ = 0.38 × (1.17 × ISF_mg/dL_) − 5.60.

**Discussion:**

Our results show that the new equations are likely to provide better estimates of HbA1c levels than the standard model at the population level, which is especially suited for clinical epidemiology in Indian populations.

## 1 Introduction

Type 2 diabetes mellitus (T2D) is one of the most common metabolic disorders in India. Understanding the metabolic pathways and mechanisms involved in the development of T2D in patients play an important role in its diagnosis and treatment. Traditionally, it involves measuring the fasting blood glucose concentration (FBG) and postprandial blood glucose concentration (PPBG). Since the late 1970s, there have been reports of a correlation between HbA1c and blood glucose concentration (BG), and that HbA1c could be a useful tool for long-term BG control. Gabbay et al. (1) studied the correlation between HbA1a, HbA1b, and HbA1c with 24-hour urinary glucose concentration collected over periods of 1, 2, and 3 months for 220 diabetic patients and suggested that glycosylated hemoglobin could act as a good index for long-term BG levels in people with T2D. Santiago et al. (2) further studied the correlation between HbA1c and PPBG. Clarke et al. (3) showed that HbA1c is correlated with aBG over 2 months, and therefore, is a good index for aBG and is a useful tool for understanding the quality of BG control in a patient. Lecomte et al. (4) also confirmed in a group of 138 patients that HbA1c is a good index for BG control. Distiller (5) compared the efficacy of PPBG and HbA1c as indices for BG control and showed that HbA1c is a significantly better index.

There is a plethora of other new metrics being developed to understand the glycemic state of the patient, such as time in range (TIR). HbA1c is one of the most reliable metrics for understanding long-term BG changes in a patient. Therefore, accurate experimental methods (6) have been developed to measure HbA1c levels. However, with the development of flash glucose monitoring (FGM) and eventually CGM technologies, clinicians now have access to many consecutive interstitial fluid glucose concentration (ISF) measurements (CGM traces). This encouraged the development of methods for estimating metrics such as FGM, PPBG, TIR, and HbA1c from the CGM traces.

Sikaris (7) showed that although for a single measurement HbA1c and BG had been shown to be correlated, including multiple measurements like CGM traces improved the correlation further. They concluded that not only was estimating HbA1c from the HbA1c–BG relationship viable, but also that it would become the standard method of estimating HbA1c. Mazze (8) also showed that BG from self-monitoring blood glucose (SMBG) and CGM traces were highly correlated, and that the HbA1c estimates obtained using them were not significantly different, although different patterns of SMBG and CGM traces could produce the same HbA1c. This suggests that HbA1c is a metric that can be reliably estimated. Nathan et al. (9) used linear regression to estimate aBG from HbA1c levels at the population level. This relationship has been used to develop a method for estimating HbA1c levels from aBG. This method was adopted as the standard for obtaining HbA1c estimates (10). This method was also used to estimate HbA1c values using the Abbot Libre FreeStyle Pro device for the CGM report generated by the device.

In recent years, after Nathan et al. (9) published their method, many similar methods have been developed for estimating HbA1c. Kovatchev et al. (11) provided a dynamic method for accurately estimating average HbA1c using regular SMBG readings for T2D patients. The method was later validated in patients with type 1 diabetes (T1D) and showed similar performance (12). Beck et al. (13) showed that experimentally measured HbA1c alone cannot be reliably used as a metric for glycemic control in an individual. They suggested that the glucose profile from the CGM trace and aBG calculated from the CGM trace were also considered. They also provided a method for estimating HbA1c from a given CGM trace and suggested that estimated HbA1c should also be considered as a metric for an individual’s glycemic control. Fan et al. (14) had established a relationship between HbA1c and FBG and PPBG which are both categorized as SMBG. They also provided a method for estimating HbA1c but also showed that FBG and HbA1c levels are strongly correlated.

Bergenstal et al. (15) renamed the estimated HbA1c as the glucose management indicator (GMI), a metric for glycemic control and management. They also provided a new method for estimating GMI from a given CGM trace. The model was then validated by Leelarathna et al. (16) using guardian 3 and navigator 2 sensor data. Perlman et al. (17) however showed that there can be a substantial difference between experimentally measured HbA1c and GMI values for T1D patients especially, with patients having advanced chronic kidney disease. Shah et al. (18) also showed that it does not correlate well with HbA1c for non-diabetic patients. Estimated HbA1c is increasingly being replaced by GMI, which is used as a metric for glycemic control. Therefore, attempts to improve GMI to closely reflect HbA1c levels and be a reliable metric for glycemic control are an active field of research.

Recently, Oriot and Hermans (19) showed that HbA1c values were overestimated using Nathan’s equation from CGM traces obtained using the Free Style Libre device for T1D patients. This contrasts with Hu et al. (20), who showed that HbA1c estimated using Nathan’s equation on CGM traces obtained by FreeStyle Libre underestimated the experimental HbA1c values. Hu et al. (20) also produced a total of seven models, based on linear and nonlinear regression analysis for estimating HbA1c values from a given CGM trace. These reliability issues of GMI or estimated HbA1c indicate that there is still a need for a new method for estimating HbA1c from a given CGM trace for an individual that works for all pre-diabetic, diabetic, and non-diabetic groups. Xu et al. (21) suggested a kinetic model for estimating HbA1c and showed that it provides a highly accurate estimate of HbA1c. He also improved the kinetic model to include the life-cycle of the red blood cells (RBC) containing the HbA1c molecules (22).

We show that the HbA1c estimates obtained using Nathan’s equation are not statistically reliable for the Indian population, and we provide two new methods for estimating HbA1c.

## 2 Materials and methods

### 2.1 Subject recruitment and measurement of blood biochemical parameters

A CGM dataset containing traces of 50 participants was collected at the Primary Care Health Centre, Savitribai Phule Pune University, Pune. For each participant, a FreeStyle Libre Pro CGM sensor (Abbott, UK) was inserted subcutaneously on the back of the upper arm by Dr. Shashikant Dudhgaonkar at the Health Centre, Savitribai Phule Pune University, Pune, India, between July 2021 and September 2021. This factory-calibrated glucose sensor recorded subcutaneous ISF every 15 min for 14 days. All participants were advised to continue their normal diet and exercise routine. On the 14th day, the CGM device was removed, and the data were downloaded and analyzed using FreeStyle Libre Pro software. The CGM devices were provided to the participants through the Rastriya Ucchattar Shiksha Abhiyan (RUSA) grant from Savitribai Phule Pune University. We refer to this dataset as the Pune-2021 dataset.

The CGM data collected in the Pune-2021 dataset were then filtered into two sets: a *data-sufficient* Pune-2021 dataset and a *data-insufficient* Pune-2021 dataset in the following way. Data sufficiency was checked according to (i) the number of days the sensor was active, and (ii) the percentage of measurements recorded, as suggested by Danne et al. (10): From the measurement ID provided in the CGM trace data file, the number of ISF measurements, *N*, recorded by the device was calculated. The timestamps provided in the CGM trace were used to calculate the effective number of days, *n_d_* for which the CGM device was active. However, this number was rounded off to the nearest integer using the round function provided by the NumPy package (23). The percentage of measurements recorded by the device was calculated using Δ*t_m_*, which is the time difference in seconds between the first and last readings. Δ*t_m_* was used to calculate the total number of readings recorded by the device as $N_{total}=\lfloor \frac{△t_{m}}{60\times 15}\rfloor +1$ . The percentage of measurements was calculated as *N_p_* = 100 × *N/N*_total_. If *n_d_* ≥14 and *N_p_* ≥70% for a given CGM trace, we categorized the CGM trace as data-sufficient; otherwise, it was categorized as data-insufficient.

After the data-insufficient CGM traces were filtered out, 12 pre-diabetic, 13 diabetic, and 14 non-diabetic CGM traces remained and were categorized as data-sufficient.

A second dataset of 78 CGM traces along with their HbA1c levels (by HPLC) was collected by Dr. K. M. Shelgikar at the Tertiary Care Center, Joshi Hospital, Shivaji Nagar, Pune from 2018 to 2020. Data were collected as part of routine patient care and anonymized for analysis. This dataset is referred to as the Joshi-2018 dataset. Similar to the Pune-2021 dataset, the Joshi-2018 dataset was filtered as data-sufficient and data-insufficient subsets. After filtering out the data-insufficient CGM traces from the Joshi-2018 dataset, only 73 CGM traces were considered as data sufficient.

The complete CGM-dataset, including both the Pune-2021 dataset and the Joshi-2018 dataset, contained the CGM traces and the corresponding HbA1c levels of 128 participants, 15 of whom were pre-diabetic, 94 were diabetic, and 19 were non-diabetic. The data-sufficient subset of the CGM-dataset contained 112 CGM traces, of which 12 were pre-diabetic, 86 were diabetic, and 14 were non-diabetic. A sample of 32 data-sufficient CGM traces and their corresponding HbA1c measurements was separated as a test set for validation purposes; the remaining 80 data-sufficient CGM traces were grouped as the training CGM-dataset. The complete CGM-dataset including the data-insufficient CGM traces was used to validate the HbA1c estimates obtained using the Nathan model (9) but only the data-sufficient CGM traces of the training CGM-dataset were used to construct our models, which were then validated using the data-sufficient CGM traces of the test CGM-dataset.

### 2.2 Comparing Nathan HbA1c estimates with experimentally measured HbA1c

Nathan et al. (9) collected a dataset of 2,700 glucose measurements from 268 T1D patients, 159 T2D patients, and 80 non-diabetic participants. Their dataset contained CGM traces and finger-stick measurements that were collected as different measures of glycemia.

The ISF measurements were scaled by a factor of 1.05 to estimate the corresponding BG. aBG was calculated by taking the weighted average of all the blood glucose concentration measurements collected. All measurements in a day were given equal weights, which were inversely proportional to the number of measurements taken on that day. The aBG was calculated by taking the mean of all measurements, giving the measurements on each day an equal weight. The expression to obtain the aBG is 


(1) $$\text{aBG}=\frac{1}{\left(m_{1}+m_{2}\right)}\{\sum_{i=1}^{i=m_{1}}(\frac{1}{n_{1,i}}){\text{BG}}_{i}+\sum_{i=1}^{i=m_{2}}(\frac{1}{n_{2,i}})(1.05\times {\text{ISF}}_{i})\},$$

where aBG represents the average blood glucose concentration, BG_i_ is the *i*th SMBG measurements, ISF_i_ is the *i*th CGM measurement, *m*_1_ and *m*_2_ are the number of SMBG and CGM measurements respectively, *n*_1_ *_,i_* is the number of SMBG measurements taken on the day *BG_i_* was taken, and *n*_2_ *_,i_* is the number of CGM measurements taken on the day *ISF_i_* was taken.

A linear regression analysis was performed by Nathan et al. (9) taking the calculated aBG as the dependent variable and the HbA1c as the independent variable and obtained this relation:


(2) $$aBG_{mg/dL}=28.7\times HbA1c_{\%}-46.7,$$

(2) can also be written as:


(3) $$\text{HbA}1c_{\%}=\frac{1}{28.7}\{46.7+(1.05\times {\text{aISF}}_{mg/dL})\},$$

(4) $$=1.627+0.035\times (1.05\times {\text{aISF}}_{mg/dL}),$$

(5) $$\text{HbA}1c_{mmol/mol}=0.38\times (1.05\times {\text{aISF}}_{mg/dL})-5.60,$$

to relate HbA1c and aISF directly.

We used a paired t-test to verify whether the two groups, that is, the experimentally measured HbA1c from the CGM-dataset and the corresponding HbA1c calculated using the Nathan model Eq. (5), and Eq. (1), are statistically indistinguishable. Calculations were performed using the ttest_rel function of the stats module of the SciPy package (24). Similarly, a paired t-test was performed with only the data sufficient (including both the training and test datasets) CGM traces from the CGM dataset.

### 2.3 Direct model

To directly construct a model between aISF and HbA1c, we assumed a linear relationship and performed a regression analysis. Note that the aISF here is an equally weighted average of all the ISF measurements in a given CGM trace,


(6) $${\text{aISF}}_{mg/dL}=\frac{1}{N}\sum_{i=1}^{i=N}{\text{ISF}}_{i,mg/dL},$$

where aISF_mg_ *_/_*_dL_ represents the calculated aISF in mg*/*dL, ISF_i_ *, *_mg_ *_/_*_dL_ represents the *i*th ISF measurement from the given CGM trace in mg*/*dL and *N* represents the total number of measurements in the given CGM trace. The linear regression equation for the direct model is 


(7) $$HbA1c_{mmol/mol}=\beta_{1}\times aISF_{mg/dL}+\beta_{0},$$

where aISF_mg_ *_/_*_dL_ represents the aISF in mg*/*dL, HbA1c_mmol_ *_/_*_mol_ represents the HbA1c in mmol*/*mol, *β*_1_ the slope in mmoldL*/*(molmg) and *β*_0_ the intercept in mmol*/*mol.

We obtained the ordinary least square (OLS) estimates ${\hat{\beta }}_{0}$ and, ${\hat{\beta }}_{1}$ of the parameters *β*_0_ and, *β*_1_. We also calculated the 95% confidence interval for ${\hat{\beta }}_{0}$ and ${\hat{\beta }}_{1}$ along with 95% confidence interval and the 95% prediction interval of HbA1c for any given aISF. This analysis was performed using the LinearRegression function from the linear_model module of the scikit-learn package (25). However, the confidence and prediction intervals were calculated using the standard OLS solution formulae.

A paired t-test was then performed on the HbA1c estimated using the direct model and the experimental values for the data-sufficient CGM traces from the test CGM-dataset to verify whether the HbA1c estimates obtained using ${\hat{\beta }}_{0}$ and ${\hat{\beta }}_{1}$ were statistically indistinguishable from the experimental HbA1c value at the population level. The t-test was performed using the ttest_rel function of the stats module of the SciPy package.

We also used the training dataset of CGM traces and calculated the 5-fold cross validation root mean squared error (RMSE) to validate the 95% confidence interval for the direct model.

### 2.4 Adapted Nathan model

In *Section 2.3*, we constructed a linear model for estimating HbA1c from the aISF calculated from a given CGM trace. Although such a relationship, if reliable, can be valuable, it requires us to base our HbA1c estimates on the ISF values. Traditionally, however, for the diagnosis of T2D and analysis of the glycemic state of an individual, various metrics such as FBG, PPBG, and HbA1c have always been based on BG. The current CGM devices, however, report ISF readings, and therefore, to use these CGM traces with our current diagnostic methods, it is important to develop a reliable method for converting the ISF readings to their corresponding BG readings. The ISF measurements in the CGM traces of the dataset used by Nathan et al. (9) were scaled to their BG values using a scaling factor of 1.05. We suspected that obtaining a better estimate of this scaling factor would improve HbA1c estimates.

Therefore, we constructed a linear model for estimating HbA1c from the calculated aISF [aISF was constructed using Eq. (6)] via the aBG. We considered a model in which we estimated aBG by scaling aISF by a factor of *ω* and used Eq. (2) to obtain the estimate of HbA1c. This model represented by Eq (8). is 


(8) $$HbA1c_{mmol/mol}=\;0.38\times \left(\omega \times aISF_{mg/dL}\right)-5.60,$$

where aISF_mg_ *_/_*_dL_ represents the aISF in mg*/*dL, HbA1c_mmol_ *_/_*_mol_ represents the HbA1c in mmol*/*mol and, *ω* the scaling factor. Now, Eq. (8) can also be written as:


(9) $$\frac{HbA1c_{mmol/mol}+5.60}{0.38}=\left(\omega \times aISF_{mg/dL}\right),$$

The OLS solutions for the estimate $\hat{\omega }$ , of the coefficient *ω* are the same for both Eqs. (8) and (9).

We obtain the OLS estimate $\hat{\omega }$ using Eq. (9), where we took the calculated aISF_mg_ *_/_*_dL_ as the independent variable and the transformed experimental HbA1c values, $\frac{HbA1c_{mmol/mol}+5.60}{0.38}$ , as the dependent variable. The analysis was performed using the LinearRegression function from the linear_model module of the scikit-learn package. Data-sufficient CGM traces and their corresponding HbA1c values from the training CGM dataset were used for this analysis. We calculated the 95% confidence interval for $\hat{\omega }$ , the 95% confidence interval and 95% prediction interval for estimated HbA1c corresponding to an aISF calculated from any CGM trace. These intervals were calculated using standard OLS solution formulae for constrained linear regression.

A paired t-test was performed with the HbA1c estimates made using the adapted Nathan model and the experimentally measured HbA1c values for the data-sufficient traces of the test CGM-dataset, using the ttest_rel function from the stats module of the SciPy package to confirm that the HbA1c estimates from the adapted Nathan model were not significantly different from the experimental HbA1c values at the population level.

Finally, using the training CGM-datset a 5-fold cross validation RMSE was calculated for the adapted Nathan model to validate the reliability of the 95% confidence intervals of the adapted Nathan model.

## 3 Results

**Figure 1:**
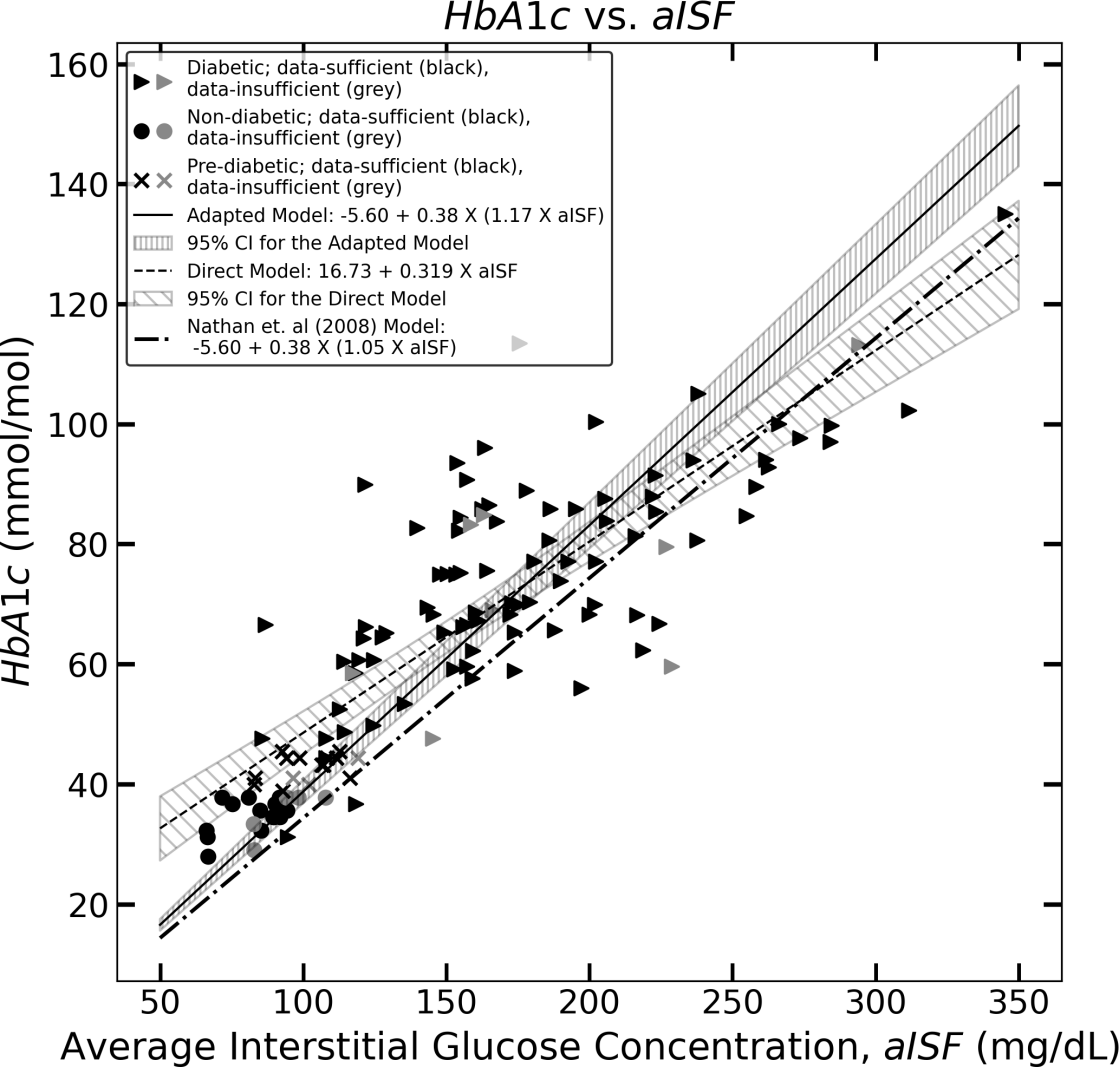
The figure represents the experimentally measured HbA1c and aISF values (calculated as described in *Section 2.3*) of the CGM dataset. The pre-diabetic participants are represented by crosses, diabetic participants are represented by solid triangles and non-diabetic participants are represented by solid circles. The scatter points representing participants with a data-sufficient CGM trace [according to Danne et al. (10)] are colored black, whereas the participants with a data-insufficient CGM trace are colored gray. The solid black line and the corresponding hatched region represent Eq. (11), which the 95% confidence interval, and the dashed line along with its corresponding hatched region, represents Eq. (10) and its corresponding 95% confidence interval. The dotted dash line represents Eq. (5).

**Figure 2:**
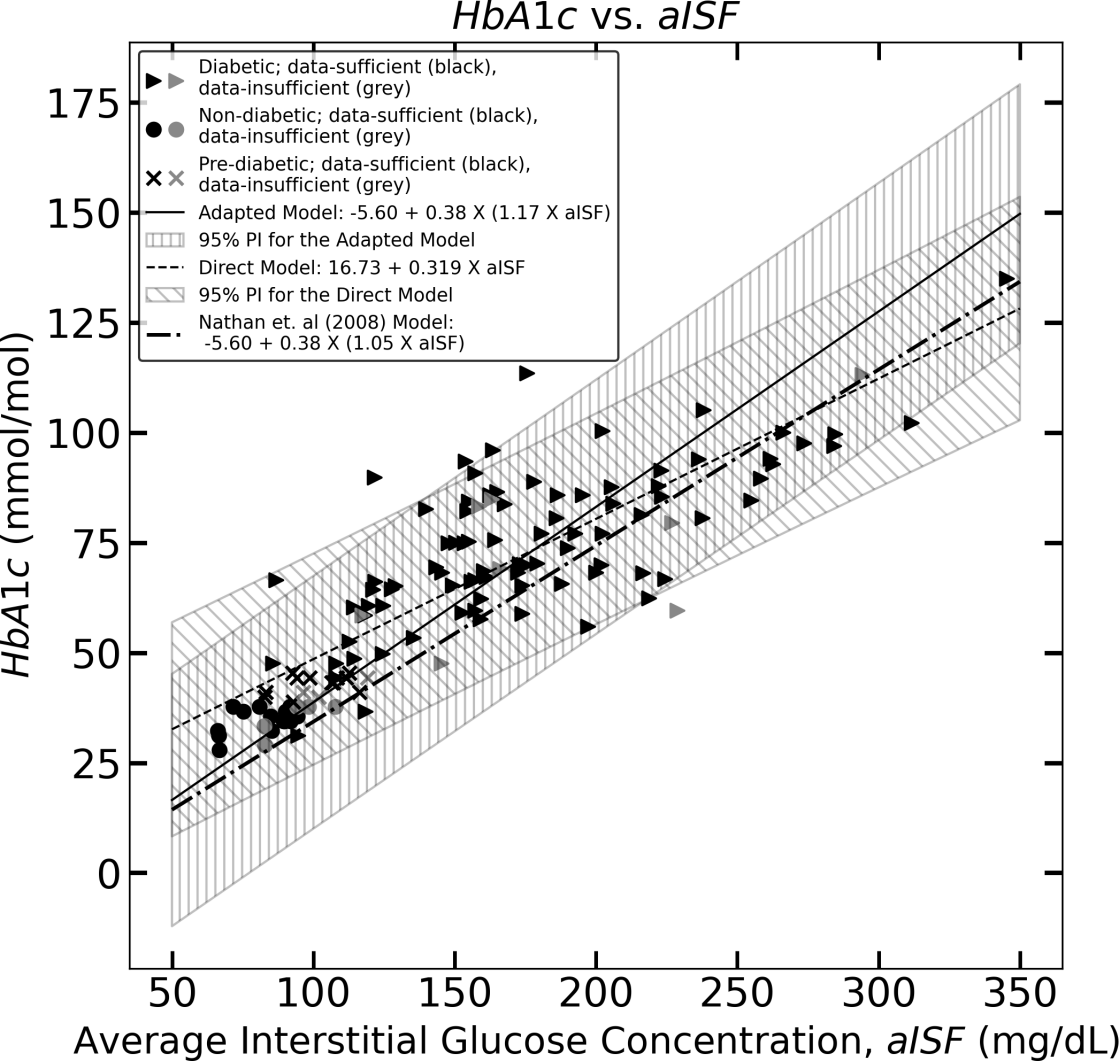
The figure represents the experimentally measured HbA1c and aISF values (calculated as described in *Section 2.3*) of the CGM dataset. The pre-diabetic participants are represented by crosses, diabetic participants are represented by solid triangles and, non-diabetic participants are represented by solid circles. The scatter points representing participants with a data-sufficient CGM trace [according to Danne et al. (10)] are colored black, whereas the participants with a data-insufficient CGM trace are colored gray. The solid black line and the corresponding hatched region represent Eq. (11), which the 95% prediction interval, and the dashed line, along with its corresponding hatched region, represents Eq. (10) and its corresponding 95% prediction interval. The dotted dash line represents Eq. (5).

We show that the mean of the estimates provided by the standard Nathan et al. (9) method for HbA1c at the population level is not statistically reliable with respect to the experimental HbA1c values for an Indian population. Next, we provide the results for the direct model and the adapted Nathan model based on OLS linear regression for estimating HbA1c from a given CGM trace. We provide the 95% confidence interval for the two models and the 95% prediction intervals for the HbA1c estimates of these two models, which are visualized in **Figures 1**, **2**, showing the three models for estimating HbA1c along with the 95% confidence interval (**Figure 1**) and the 95% prediction interval (**Figure 2**). We also provide a user-friendly web app for academic use, CGM Analyzer [version 0.1] (https://digimed.acads.iiserpune.ac.in/fgm-tools), created using MATLAB R2022a. The HbA1c estimates along with their 95% prediction intervals can be calculated for a given CGM trace.

### 3.1 Coefficient estimates for the direct model

The paired t-test was performed using the CGM traces of the complete CGM-dataset between the experimental HbA1c values and the corresponding Nathan HbA1c estimates calculated using Eqs (1). and (5) generated a p-value<0.001. Similarly, a paired t-test with data-sufficient CGM traces from the CGM-dataset also generated a p-value of<0.001. Considering *α* = 0.05, the Nathan model-estimated HbA1c values, both for data-sufficient and data-insufficient CGM traces, were significantly different from the corresponding experimentally measured HbA1c values for the Indian population.

This led us to construct a direct model for estimating HbA1c levels from aISF. We performed a linear regression analysis to establish a relationship between aISF and HbA1c using the data-sufficient CGM traces of the training CGM dataset. We obtained an estimate of the coefficient *β*_0_ of the model, Eq. (7), ${\hat{\beta }}_{0}=16.73\;mmol/mol$ , with a 95% confidence interval of [9.39 mmol*/*mol, 24.07 mmol*/*mol] and an estimate for *β*_1_, ${\hat{\beta }}_{1}=0.319\;mmol\;dL/\left(molmg\right)$ with a 95% confidence interval of [0.274 mmol dL*/*(molmg), 0.363 mmol dL*/*(molmg)]. The analysis yielded Eq. (10), with an *R* ^2^ = 0.726 and a *p*-*value <*0.01. Therefore, the direct model, Eq. (7) with the estimates ${\hat{\beta }}_{0}$ and ${\hat{\beta }}_{1}$ is given by 


(10) $$HbA1c_{mmol/mol}=\;0.319\;\times \;aISF_{mg/dL}\;+\;16.73,$$

where aISF_mg_ *_/_*_dL_ represents the aISF in mg*/*dL, and HbA1c_mmol_ *_/_*_mol_ represents HbA1c in mmol*/*mol. **Figure 1**, shows Eq. (10) as the black dashed line along with the 95% confidence interval for HbA1c_mmol_ *_/_*_mol_, corresponding to any aISF_mg_ *_/_*_dL_ calculated from a given CGM trace. The formulae for obtaining the 95% confidence and prediction interval for any HbA1c estimate are provided in the **Supplementary Material**. The 95% prediction interval width calculated for the HbA1c estimates was on the order of 48.50 mmol*/*mol.

### 3.2 Coefficient estimates of the adapted Nathan model

We constructed a direct model, given by Eq. (10) to estimate HbA1c from the aISF for any given CGM trace. However, we suspect that the model described in Eq. (8), where HbA1c was estimated from a scaled aISF value using Eq. (2) would provide better estimates of HbA1c levels. The OLS solution for linear regression analysis using Eq. (9), while keeping the intercept zero, would provide us with an estimate of the scaling factor for obtaining aBG from aISF.

The estimation of the scaling factor in Eq. (9), $\hat{\omega }$ , obtained using the LinearRegression function of the scykit-learn package with the intercept set to zero, on the data-sufficient training CGM-dataset, is $\hat{\omega }\;=\;1.17$ , with a 95% confidence interval (1.12, 1.22). The analysis yielded an *R* ^2^ = 0.595 and, a *p*-*value<*0.01. The estimate, $\hat{\omega }$ , obtained using the analytical solution for obtaining the OLS estimate of *ω* from Eq. (8) yields an identical result. The equation for obtaining HbA1c estimates using the adapted Nathan model is represented by Eq. (11) below 


(11) $$HbA1c_{mmol/dL}=\;0.38\;\times \;\left(1.17\;\times \;aISF_{mg/dL}\right)-5.60,$$

where aISF_mg_ *_/_*_dL_ represents the aISF in mg*/*dL, and HbA1c_mmol_ *_/_*_mol_ represents HbA1c in mmol*/*mol. **Figure 1** shows Eq. (11) as a solid black line, along with the 95% confidence interval for HbA1c_mmol_ *_/_*_mol_ corresponding to any aISF_mg_ *_/_*_dL_. The formulae for obtaining the confidence and prediction intervals for any HbA1c value estimated using Eq. (11) are provided in the **Supplementary Material**. The 95% prediction interval width calculated for the HbA1c estimates are in the order of 57.87 mmol*/*mol.

### 3.3 Validation of the direct and adapted models

In *Sections 3.1 and 3.2*, we constructed two models for estimating HbA1c from the aISF calculated from any given CGM trace. We then constructed 95% prediction intervals for HbA1c estimates calculated using the models. The formulae for constructing the prediction interval corresponding to any estimated HbA1c level for both models are provided in the **Supplementary Material**.

A paired t-test performed between the experimentally measured HbA1c from the test CGM-dataset and the HbA1c estimates obtained from their corresponding CGM trace using the direct model generates a p-value of 0.643 and using the adapted Nathan model it generates a p-value of 0.715. This indicates that at the population level, the HbA1c estimates for an independent sample of CGM traces were statistically (*α* = 0.05) indistinguishable from the experimental HbA1c. The 5-fold cross validation root mean squared error (RMSE) for the HbA1c estimates obtained using the direct model on the training CGM-datasets is 11.9 mmol*/*mol and for the estimates obtained using the adapted Nathan model it is 14.3 mmol*/*mol. The 95% confidence interval for the direct model was on the order of 9.48 mmol*/*mol and for the adapted Nathan model the 95% confidence interval was on the order of 7.70 mmol*/*mol.

A t-test performed using the Nathan model HbA1c estimates for the test CGM-dataset generated a p-value<0.01, indicating that at the population level, the Nathan model HbA1c estimates were statistically different from the experimental value (taking *α* = 0.05).

## 4 Discussion

The development of CGM technology provides a large number of glucose concentration measurements. This provides a great opportunity to study the glucose dynamics and glycemic state of an individual. The current CGM devices, however, only provide ISF measurements, while traditionally it has been the norm to study glucose dynamics with BG measurements. Therefore, a large bulk of our understanding of glucose dynamics, glycemic states, and metabolic diseases, such as diabetes, is based on BG values. To use the CGM traces provided by these devices, it is important to reliably estimate the corresponding BG, especially HbA1c, from any given CGM trace. Typically, regression estimates are used to relate the average glucose level from the CGM to HbA1c. Bailey et al. (26) showed that a 7-day CGM trace provides a satisfactory estimate of GMI or estimated HbA1c comparable to estimates obtained from 14-day CGM.

The analyses conducted by Nathan et al. (9), Hu et al. (20), Bergenstal et al. (15), and Xu et al. (21) used large CGM trace datasets and their corresponding HbA1c values. While these are important estimates, it is equally important to ask if these models continue to be applicable to different populations. Indeed, it has been shown that regression equations vary with ethnicity; for instance, Hu et al. (20) and Oriot and Hermans (19) cite over- or underestimation relative to the Nathan model. To the best of our knowledge, no major study has validated these estimates in an Indian population.

We used a dataset of 128 CGM traces collected from an Indian population, sorted to use only data-sufficient CGM traces to construct models suitable for this population. We showed that the standard method of estimating HbA1c using Nathan’s equation does not provide a statistically reliable estimate. Therefore, we suggest two new methods for estimating HbA1c that are better suited to the Indian population. The direct method for estimating HbA1c from ISF values, as described in *Section 2.3*, provides an estimate along with a 95% confidence and prediction interval for the estimate given an aISF value. The mean HbA1c estimates provided by the direct model were statistically indistinguishable from the mean experimental HbA1c measurement for the data-sufficient test CGM-dataset. Furthermore, we suspected that the inclusion of an improved method of estimating BG from ISF could improve the estimates provided by Eq. (5). Therefore, in *Section 3.2*, we constructed a new linear model for estimating BG from ISF using linear regression. The mean HbA1c estimates provided by this method were indistinguishable from the mean experimentally measured HbA1c values of the data-sufficient test CGM-dataset. However, the 95% prediction interval was large. We showed that the mean HbA1c estimates obtained using these two models, the direct model, and the adapted Nathan model, were not significantly different from the mean experimental HbA1c. However, the mean experimental HbA1c level was significantly different from the mean estimates provided by the Nathan model at the population level.

From the analysis of the model performance on the test CGM-dataset, we can conclude that although our models for estimating HbA1c provide a wide 95% prediction interval, which includes the HbA1c estimates obtained using the Nathan model, the mean HbA1c estimates provided by our models at the population level are statistically indistinguishable from the mean experimental HbA1c values, unlike the HbA1c estimates obtained using the Nathan model. This shows that the direct and adapted Nathan models can provide a more reliable HbA1c estimate than the Nathan model can. Such estimates are valuable at the population level, as in clinical epidemiological studies.

The strength of thsi study is that it is the first investigation of its kind in an Indian population. Furthermore, we outline that there are subtleties in the estimation procedure; depending on the question of interest, these lead to alternate formulations of the problem. We applied both approaches to the same dataset, which made it easier to compare the two methods. The weakness of our study is that the dataset was limited, and the results should be seen as prospective. We hope that future studies will test these hypotheses with greater statistical power.

Because the computed prediction intervals are rather wide, we claim that none of the models described above are suitable for estimating HbA1c in individuals with (clinical) reliability. This raises a deeper question: Can individual HbA1c estimates be obtained using only aISF values calculated from a CGM trace? Or does it require knowledge of some additional information regarding the individual not contained in their CGM? That is, it remains an open question although ISF and BG are highly correlated with HbA1c, why are the models unable to provide tighter estimates of HbA1c from aISF or aBG values alone?

## Data availability statement

The raw data supporting the conclusions of this article will be made available by the authors, without undue reservation.

## Ethics statement

The study protocol for the Pune-2021 dataset was approved by the Institutional Ethical Committee (IEC) of Savitribai Phule Pune University, Pune, India (SPPU/IEC/2020/102). The study protocol for the Joshi-2018 dataset was approved by the IEC of Maharashtra Medical Research Society (ECR/311/Inst/MH/2013/RR-19; Dated 14 March 2023). Informed consent from all participants was collected prior to their participation in the studies. The data analysis was reviewed and approved by the Institutional Human Ethics Committee (IHEC) of IISER Pune (IHEC/Admin/2021/015).

## Author contributions

SM: Software, Visualization, Writing – original draft, Methodology. SK: Data curation, Writing – review & editing, Investigation. SD: Data curation, Writing – review & editing, Investigation. KS: Data curation, Writing – review & editing, Investigation. SG: Data curation, Funding acquisition, Project administration, Writing – review & editing, Conceptualization, Investigation, Supervision. PG: Supervision, Writing – original draft, Writing – review & editing, Methodology, Conceptualization.

## References

[1] Gabbay KH Hasty K Breslow JL Ellison RC Bunn HF Gallop PM. Glycosylated hemoglobins and long-term blood glucose control in diabetes mellitus. J Clin Endocrinol Metab (1977) 44:859–64. doi: 10.1210/jcem-44-5-859 858776

[2] Santiago JV Davis J Fisher F. Hemoglobin a1c levels in a diabetes detection program. J Clin Endocrinol Metab (1978) 47:578–80. doi: 10.1210/jcem-47-3-578 263311

[3] Clarke J Passa P Canivet J. Hba1c: need of its dosage in diabetics (author’s transl). La Nouvelle Presse Medicale (1979) 8:513–7. 461162

[4] Lecomte M Schoos R Schoos-Barbette S Luyckx A Lambotte C Lefebvre P. Hemoglobin a1c and diabetes control (author’s transl). Diabete Metabolisme (1979) 5:57–61. 446835

[5] Distiller LA Zail SS. The use of glycosylated haemoglobin measurements in the control of the diabetic patient. South Afr Med J (1979) 55:335–7. 441895

[6] Davis J Mcdonald JM. Jarett L. A high-performance liquid chromatography method for hemoglobin a1c. Diabetes (1978) 27:102–7. doi: 10.2337/diab.27.2.102 624438

[7] Sikaris K. The correlation of hemoglobin a1c to blood glucose. J Diabetes Sci Technol (2009) 3:429–38. doi: 10.1177/193229680900300305 PMC2769865 20144279

[8] Mazze R. The future of self-monitored blood glucose: mean blood glucose versus glycosylated hemoglobin. Diabetes Technol Ther (2008) 10:S–93. doi: 10.1089/dia.2008.0006

[9] Nathan DM Kuenen J Borg R Zheng H Schoenfeld D Heine RJ. Translating the a1c assay into estimated average glucose values. Diabetes Care (2008) 31:1473–8. doi: 10.2337/dc08-0545 PMC2742903 18540046

[10] Danne T Nimri R Battelino T Bergenstal RM Close KL DeVries JH. International consensus on use of continuous glucose monitoring. Diabetes Care (2017) 40:1631–40. doi: 10.2337/dc17-1600 PMC6467165 29162583

[11] Kovatchev BP Flacke F Sieber J Breton MD. Accuracy and robustness of dynamical tracking of average glycemia (a1c) to provide real-time estimation of hemoglobin a1c using routine self-monitored blood glucose data. Diabetes Technol Ther (2014) 16:303–9. doi: 10.1089/dia.2013.0224 PMC3997127 24299302

[12] Kovatchev BP Breton MD. Hemoglobin a1c and self-monitored average glucose: validation of the dynamical tracking ea1c algorithm in type 1 diabetes. J Diabetes Sci Technol (2016) 10:330–5. doi: 10.1177/1932296815608870 PMC4773966 26553023

[13] Beck RW Connor CG Mullen DM Wesley DM Bergenstal RM. The fallacy of average: how using hba1c alone to assess glycemic control can be misleading. Diabetes Care (2017) 40:994–9. doi: 10.2337/dc17-0636 PMC5521971 28733374

[14] Fan W Zheng H Wei N Nathan DM. Estimating hba1c from timed self-monitored blood glucose values. Diabetes Res Clin Pract (2018) 141:56–61. doi: 10.1016/j.diabres.2018.04.023 29673846

[15] Bergenstal RM Beck RW Close KL Grunberger G Sacks DB Kowalski A. Glucose management indicator (gmi): a new term for estimating a1c from continuous glucose monitoring. Diabetes Care (2018) 41:2275–80. doi: 10.2337/dc18-1581 PMC6196826 30224348

[16] Leelarathna L Beck RW Bergenstal RM Thabit H Hovorka R. Glucose management indicator (gmi): insights and validation using guardian 3 and navigator 2 sensor data. Diabetes Care (2019) 42:e60–1. doi: 10.2337/dc18-2479 30728221

[17] Perlman JE Gooley TA McNulty B Meyers J Hirsch IB. Hba1c and glucose management indicator discordance: a real-world analysis. Diabetes Technol Ther (2021) 23:253–8. doi: 10.1089/dia.2020.0501 PMC8255314 33253015

[18] Shah VN Vigers T Pyle L Calhoun P Bergenstal RM. Discordance between glucose management indicator and glycated hemoglobin in people without diabetes. Diabetes Technol Ther (2023) 25:324–8. doi: 10.1089/dia.2022.0544 36790875

[19] Oriot P Hermans MP. “mind the gap please”: estimated vs. measured a1c from continuous measurement of interstitial glucose over a 3-month period in patients with type 1 diabetes. Acta Clinica Belgica (2020) 75:109–15. doi: 10.1080/17843286.2018.1561780 30596337

[20] Hu Y Shen Y Yan R Li F Ding B Wang H. Relationship between estimated glycosylated hemoglobin using flash glucose monitoring and actual measured glycosylated hemoglobin in a chinese population. Diabetes Ther (2020) 11:2019–27. doi: 10.1007/s13300-020-00879-x PMC7434821 32696267

[21] Xu Y Dunn TC Ajjan RA. A kinetic model for glucose levels and hemoglobin a1c provides a novel tool for individualized diabetes management. J Diabetes Sci Technol (2021) 15:294–302. doi: 10.1177/1932296819897613 31910672 PMC8256073

[22] Xu Y Bergenstal RM Dunn TC Ajjan RA. Addressing shortfalls of laboratory hba1c using a model that incorporates red cell lifespan. Elife (2021) 10:e69456. doi: 10.7554/eLife.69456 34515636 PMC8437432

[23] Harris CR Millman KJ van der Walt SJ Gommers R Virtanen P Cournapeau D. Array programming with numPy. Nature (2020) 585:357–62. doi: 10.1038/s41586-020-2649-2 PMC7759461 32939066

[24] Virtanen P Gommers R Oliphant TE Haberland M Reddy T Cournapeau D. SciPy 1.0: fundamental algorithms for scientific computing in python. Nat Methods (2020) 17:261–72. doi: 10.1038/s41592-019-0686-2 PMC7056644 32015543

[25] Pedregosa F Varoquaux G Gramfort A Michel V Thirion B Grisel O. Scikit-learn: machine learning in python. J Mach Learn Res (2011) 12:2825–30. doi: 10.48550/arXiv.1201.0490

[26] Bailey R Calhoun P Bergenstal RM Beck RW. Assessment of the glucose management indicator using different sampling durations. Diabetes Technol Ther (2023) 25:148–50. doi: 10.1089/dia.2022.0284 36130138

