# Supplementary material for: Evaluation of HbA1c from CGM traces in an Indian population
Source: Front Endocrinol (Lausanne). 2023 Nov 20;14:1264072. doi: 10.3389/fendo.2023.1264072 (PMC10694347; doi:10.3389/fendo.2023.1264072)
Supplement: Supplementary file 1 [file DataSheet_1.pdf]

Supplementary Material

This supplementary document provides details of the confidence and prediction intervals for glycated hemoglobin (HbA1c) for the direct or the adapted Nathan model.

1 CONFIDENCE AND PREDICTION INTERVALS FOR THE DIRECT MODEL

Given any continuous glucose monitoring (CGM) trace, $\mathrm{aISF}_{0,\ \mathrm{mg/dL}}$ and, $\mathrm{HbA1c}_{0,\ \mathrm{mmol/mol}}$ can be calculated using the equations specified for the direct model. The confidence interval for HbA1c corresponding to that CGM trace can be calculated using the standard error (SE) as $\mathrm{HbA1c}_{0,\ \mathrm{mmol/mol}} \pm \mathrm{SE_{CI}}$ where the $\mathrm{SE_{CI}}$ is (see Ross, 2004, section. 9.4.3)

$$\mathrm{SE_{CI}} = t_{1-\alpha/2,\ N-2} \times \sqrt{\left(\frac{\mathrm{SS_R}}{N-2}\right)\left\{\frac{1}{N} + \frac{\left(\mathrm{aISF}_{0,\ \mathrm{mg/dL}} - \overline{\mathrm{aISF}}_{\mathrm{mg/dL}}\right)^2}{\mathrm{S_{xx}}}\right\}}, \quad \text{(S.1)}$$

where $\mathrm{SS_R} = 11\,065.79\,\mathrm{mmol^2/mol^2}$ represents the sum of squares of the residuals for the data-sufficient training CGM-dataset, $N = 80$ represents the number of CGM traces and HbA1c pairs in the data-sufficient training CGM-dataset, $\mathrm{S_{xx}} = 288\,438.08\,\mathrm{mg^2/dL^2}$ represents the sum of squared deviations from the mean value of average interstitial fluid glucose concentration (aISF) for the data-sufficient training CGM-dataset, $\overline{\mathrm{aISF}}_{\mathrm{mg/dL}} = 155.0\,\mathrm{mg/dL}$ represents the mean $\mathrm{aISF}_{\mathrm{mg/dL}}$ for the data-sufficient training CGM-dataset and, $t_{1-\alpha/2,\ N-2}$ represents the t-statistic with α as the level of significance.

Similar to (S.1), given a CGM trace and the calculated $\mathrm{aISF}_{0,\ \mathrm{mg/dL}}$, we can construct a prediction interval for the corresponding HbA1c value using the SE as $\mathrm{HbA1c}_{0,\ \mathrm{mmol/mol}} \pm \mathrm{SE_{PI}}$ where $\mathrm{SE_{PI}}$ is given by (S.2) (see Ross, 2004, section. 9.4.4)

$$\mathrm{SE_{PI}} = t_{1-\alpha/2,\ N-2} \times \sqrt{\left(\frac{\mathrm{SS_R}}{N-2}\right)\left\{\frac{N+1}{N} + \frac{\left(\mathrm{ISF}_{0,\ \mathrm{mg/dL}} - \overline{\mathrm{aISF}}_{\mathrm{mg/dL}}\right)^2}{\mathrm{S_{xx}}}\right\}} \quad \text{(S.2)}$$

2 CONFIDENCE AND PREDICTION INTERVALS FOR THE ADAPTED NATHAN MODEL

Now, given any CGM trace, $\mathrm{aISF}_{0,\ \mathrm{mg/dL}}$ and $\mathrm{HbA}_{1\mathrm{c}0,\ \mathrm{mmol/mol}}$ can be calculated using the equations provided for the adapted Nathan model. The confidence interval for the corresponding HbA1c value can be calculated using the SE as $\mathrm{HbA1c}_{0,\ \mathrm{mmol/mol}} \pm \mathrm{SE_{CI}}$ where the $\mathrm{SE_{CI}}$ is calculated by using (S.3) (see section 2.1)

$$\mathrm{SE_{CI}} = t_{1-\alpha/2,\ N-1} \times \sqrt{\left(\frac{\mathrm{SS_R}}{N-1}\right)\left\{\frac{\mathrm{aISF}^2_{0,\ \mathrm{mg/dL}}}{\sum_{i=1}^{i=N} \mathrm{aISF}^2_{\mathrm{i},\ \mathrm{mg/dL}}}\right\}}, \quad \text{(S.3)}$$

where $\mathrm{SS_R} = 16\,343.06\,\mathrm{mmol^2/mol^2}$ represents the sum of squares of the residuals, $N = 80$ represents the number of CGM trace and HbA1c pairs present in the data-sufficient training CGM-dataset, and $\sum_{i=1}^{i=N} \mathrm{aISF}^2_{\mathrm{i},\ \mathrm{mg/dL}} = 2\,210\,500.08\,\mathrm{mg^2/dL^2}$ represents the sum of squares of the aISF values of the CGM

traces in the data-sufficient training CGM-dataset and, $t_{1-\alpha/2,\ N-1}$ represents the t-statistic with α as the level of significance.

Similarly, just just like (S.3), the prediction interval can be constructed using the SE as as $\mathrm{HbA1c}_{0,\ \mathrm{mmol/mol}} \pm \mathrm{SE_{PI}}$ where $\mathrm{SE_{PI}}$ is given by (S.4) (see section 2.1)

$$\mathrm{SE_{PI}} = t_{1-\alpha/2,\ N-1} \times \sqrt{\left(\frac{\mathrm{SS_R}}{N-1}\right)\left\{1 + \frac{\mathrm{ISF}^2_{0,\ \mathrm{mg/dL}}{}^2}{\sum_{i=1}^{i=N} \mathrm{ISF}^2_{\mathrm{i,\ mg/dL}}}\right\}} \tag{S.4}$$

2.1 Constructing the confidence and prediction interval for the adapted Nathan model

The regression equation for the adapted Nathan model, described in the manuscript is given by

$$\mathrm{HbA}_{1\mathrm{c},\ \mathrm{mmol/mol}} = 0.38 \times \left(\omega \times \mathrm{aISF_{mg/dL}}\right) - 5.60, \tag{S.5}$$

where $\mathrm{HbA}_{1\mathrm{c},\ \mathrm{mmol/mol}}$ represents the HbA1c in $\mathrm{mmol/mol}$, $\mathrm{aISF_{mg/dL}}$ represents the aISF in $\mathrm{mg/dL}$, ω represents the scaling factor required to convert aISF values to average blood glucose concentration (aBG) values. Now (S.5) can be generalized to

$$Y = \gamma_0 + \gamma_1 \times (\omega \times x) + \epsilon, \tag{S.6}$$

where Y represents the dependent variable corresponding to $\mathrm{HbA}_{1\mathrm{c},\ \mathrm{mmol/mol}}$, x the independent variable corresponding to $\mathrm{aISF_{mg/dL}}$, γ_0 and γ_1 the fixed coefficients obtained from the Nathan model, ϵ a random error with a mean of zero and ω the coeficient whose estimate, $\widehat{\omega}$ we are about to calculate, using ordinary least square (OLS) minimization. As (S.6) is not of the standard linear regression model form, we provide the calculations for obtaining the confidence interval and prediction interval. We follow a similar strategy to the calculations provided in Ross (2004, chap. 9).

2.1.1 The ordinary least square estimate of the scaling factior ω

Considering N observations, Y_i and x_i for $i \in \mathbb{N}_N$, where Y represents the dependent variable and x represents the independent variable, the OLS estimate, $\widehat{\omega}$, of ω can be calculated by mimimizing the sum of squares of the residuals, $\mathrm{SS_R}$, with respect to $\widehat{\omega}$ where $\mathrm{SS_R}$ can be written as

$$\mathrm{SS_R} = \sum_{i=1}^{i=N} \left[Y_i - \{\gamma_0 + \gamma_1 (\widehat{\omega} \times x_i)\}\right]^2 \tag{S.7}$$

Assuming $\frac{\partial}{\partial \widehat{\omega}} \mathrm{SS_R} = 0$ we have

$$\widehat{\omega} = \frac{1}{\sum_{j=1}^{j=N} x_j^2} \sum_{i=1}^{i=N} x_i \left(\frac{Y_i - \gamma_0}{\gamma_1}\right) \tag{S.8}$$

Therefore given any x_0, we can obtain an estimate of Y_0, $\widehat{Y}_0$ using

$$\widehat{Y}_0 = \gamma_0 + \gamma_1 (\widehat{\omega} \times x_0) \tag{S.9}$$

We also make an additional assumption that $\epsilon \overset{iid}{\sim} \mathcal{N}\left(0, \sigma^2\right)$, where we assume σ^2 is the variance for ϵ. This leads to

$$Y \sim \mathcal{N}\left(\gamma_0 + \gamma_1 \left(\omega \times x\right), \sigma^2\right) \tag{S.10}$$

Now using (S.8) the expectation of $\widehat{\omega}$ can be calculated as

$$\begin{aligned} E\left[\widehat{\omega}\right] &= \frac{1}{\sum_{j=1}^{j=N} x_j^2} \sum_{i=1}^{i=N} x_i \, E\left[\frac{Y_i - \gamma_0}{\gamma_1}\right] \\ &= \frac{1}{\sum_{j=1}^{j=N} x_j^2} \sum_{i=1}^{i=N} x_i \left(\frac{E\left[Y_i\right] - \gamma_0}{\gamma_1}\right) \\ &= \frac{1}{\sum_{j=1}^{j=N} x_j^2} \sum_{i=1}^{i=N} x_i \left(\frac{\gamma_0 + \gamma_1 \omega x_i - \gamma_0}{\gamma_1}\right) \\ &= \omega \end{aligned} \tag{S.11}$$

We now further calculate the variance of $\widehat{\omega}$ as

$$\begin{aligned} Var\left(\widehat{\omega}\right) &= Var\left(\frac{1}{\sum_{j=1}^{j=N} x_j^2} \sum_{i=1}^{i=N} x_i \left(\frac{Y_i - \gamma_0}{\gamma_1}\right)\right) \\ &= \frac{1}{\left(\sum_{j=1}^{j=N} x_j^2\right)^2} \sum_{i=1}^{i=N} x_i^2 \, Var\left(\frac{Y_i - \gamma_0}{\gamma_1}\right) \\ &= \frac{1}{\left(\sum_{j=1}^{j=N} x_j^2\right)^2} \sum_{i=1}^{i=N} \frac{x_i^2}{\gamma_1^2} Var\left(Y_i\right) \\ &= \frac{\sigma^2}{\gamma_1^2 \left(\sum_{j=1}^{j=N} x_j^2\right)} \end{aligned} \tag{S.12}$$

(S.11) clearly shows that $\widehat{\omega}$ is an unbiased estimator of ω and as $\widehat{\omega}$ is a linear combination of normal random variables with the mean given by (S.11) and variance by (S.12), we know $\widehat{\omega}$ is normally distributed as

$$\widehat{\omega} \sim \mathcal{N}\left(\omega, \frac{\sigma^2}{\gamma_1^2 \left(\sum_{j=1}^{j=N} x_j^2\right)}\right) \tag{S.13}$$

2.1.2 Confidence interval for dependent variable $\boldsymbol{Y}$

The expectation value of the estimate $\widehat{Y}_0$ obtained using (S.9) for any given x_0 is

$$\begin{aligned} E\left[\widehat{Y}_0\right] &= E\left[\gamma_0 + \gamma_1\left(\widehat{\omega} \times x_0\right)\right] \\ &= \gamma_0 + \gamma_1\left(E\left[\widehat{\omega}\right] \times x_0\right) \\ &= \gamma_0 + \gamma_1\left(\omega \times x_0\right) \\ &= Y_0 \end{aligned} \tag{S.14}$$

and the variance of the estimate $\widehat{Y}_0$ obtained using (S.9) for any given independent x_0 is

$$\begin{aligned} Var\left(\widehat{Y}_0\right) &= Var\left(\gamma_0 + \gamma_1\left(\widehat{\omega} \times x_0\right)\right) \\ &= Var\left(\gamma_0 + \frac{\gamma_1 x_0}{\sum_{j=1}^{j=N} x_j^2}\sum_{i=1}^{i=N} x_i\left(\frac{Y_i - \gamma_0}{\gamma_1}\right)\right) \\ &= \frac{x_0^2\sum_{i=1}^{i=N} x_i^2\, Var\left(Y_i\right)}{\left(\sum_{j=1}^{j=N} x_j^2\right)^2} \\ &= \frac{\sigma^2 x_0^2}{\sum_{j=1}^{j=N} x_j^2} \end{aligned} \tag{S.15}$$

It is clear from (S.14) that $\widehat{Y}_0$ is an unbiased estimate of Y_0 and as $\widehat{\omega}$ is normally distributed so is $\widehat{Y}$ with its mean given by (S.14) and variance by (S.15)

$$\gamma_0 + \gamma_1\left(\widehat{\omega} \times x_0\right) \sim \mathcal{N}\left(\gamma_0 + \gamma_1\left(\omega \times x_0\right),\ \frac{\sigma^2 x_0^2}{\sum_{j=1}^{j=N} x_j^2}\right) \tag{S.16}$$

Using Cochran's theorem (Cochran, 1934) we can show that the sum of squares of the residuals, $\mathrm{SS_R}$ given by (S.7), when scaled by $\frac{1}{\sigma^2}$ has a chi-square distribution with $N-1$ degrees of freedom

$$\frac{\mathrm{SS_R}}{\sigma^2} \sim \chi^2_{N-1} \tag{S.17}$$

Now, using Eq (S.16) and (S.17), we have for any level of significance, $\alpha \in (0,1)$, the $(1-\alpha)\,100\%$ confidence interval for Y_0 as $\gamma_0 + \gamma_1\left(\omega \times x_0\right) \pm \mathrm{SE_{CI}}$, where $\mathrm{SE_{CI}}$ is

$$\mathrm{SE_{CI}} = t_{1-\alpha/2,N-1}\sqrt{\frac{\mathrm{SS_R} \times x_0^2}{(N-1)\sum_{j=1}^{j=N} x_j^2}} \tag{S.18}$$

2.1.3 Prediction interval for the dependent variable $\boldsymbol{Y}$

For any given independent x_0 we can calculate $\widehat{Y}_0$ using (S.9). Using (S.16) and (S.10) we now have

$$Y_0 - \widehat{Y}_0 \sim \mathcal{N}\left(0, \sigma^2\left[1 + \frac{x_0^2}{\sum_{j=1}^{j=N} x_j^2}\right]\right) \tag{S.19}$$

Using (S.17) we can further obtain

$$\frac{Y_0 - \widehat{Y}_0}{\sqrt{\frac{\mathrm{SS_R}}{(N-1)}}\sqrt{1 + \frac{x_0^2}{\sum_{j=1}^{j=N} x_j^2}}} \sim t_{N-1} \tag{S.20}$$

Therefore, for any given independent x_o and $\alpha \in (0,1)$ level of significance the dependent variable Y has a prediction interval $\widehat{Y}_0 \pm \mathrm{SE_{PI}}$, where $\mathrm{SE_{PI}}$ is calculated using

$$\mathrm{SE_{PI}} = t_{1-\alpha/2, N-1}\sqrt{\frac{\mathrm{SS_R}}{(N-1)}}\sqrt{1 + \frac{x_0^2}{\sum_{j=1}^{j=N} x_j^2}} \tag{S.21}$$

REFERENCES

Ross S. *Introduction to Probability and Statistics for Engineers and Scientists*. Introduction to Probability and Statistics for Engineers and Scientists (Elsevier Science) (2004).

Cochran WG. The distribution of quadratic forms in a normal system, with applications to the analysis of covariance. *Mathematical Proceedings of the Cambridge Philosophical Society* **30** (1934) 178–191. doi:10.1017/S0305004100016595. Publisher: Cambridge University Press.
